# FASTK family of genes linked to cancer

**DOI:** 10.6026/97320630018206

**Published:** 2022-03-31

**Authors:** Abilasha Ramasubramanian, A Paramasivam, Pratibha Ramani

**Affiliations:** 1 Department of Oral Pathology, Saveetha Dental College and Hospitals, Saveetha Institute of Medical and Technical Sciences, Saveetha University, Poonamallee High Road, Chennai, Tamilnadu - 600077, India; 2 Department of Dental Research Cell- Blue Lab, Saveetha Dental College and Hospitals, Saveetha Institute of Medical and Technical Sciences, Saveetha University, Poonamallee High Road, Chennai, Tamilnadu - 600077, India

**Keywords:** FAST, FASTK family, cancer, mitochondrial dysfunction

## Abstract

Fas Activated Serine/Threonine Kinase (FASTK) family is a protein family encoded in the nuclear genome that spans the mitochondria and executes numerous functions, and consists of FASTK, the founding member along with 5 homologous proteins FASTKD1-5. Up regulation of FASTK family members have not only been implicated in tumour progression and invasion but also in increased resistance to chemotherapy proven by their knockdown leading to increased sensitivity to drugs. Thus, this review reports the implication of FASTK proteins in cancer and hence provides a scope to emphasise the role of these proteins in Oral Cancer.

## Background:

Cancer is characterised by a pathological breakdown in the processes that control cell proliferation, differentiation, and death 1]. It is a serious public health and economic concern, although there are significant geographical differences in the incidence of overall and specific organ cancers [2]. Oral cancer causes a significant proportion of deaths in India especially in males owing to the tobacco smoking and chewing habits, however in the Western countries oral cancer is attributed to HPV infection [2]. Oral cancer accounts for 90% of cancers arising in the head and neck region. Oral Cancer comprises cancer of the lips, tongue, gum, floor of mouth, palate, cheek mucosa, vestibule of the mouth, or retromolar area [3]. There are a plethora of genes including oncogenes, anti-apoptotic genes among others which play a major role in cancer progression. According to the latest literature, cancer is an acquired dysfunction of human cells as they precede to neoplastic growth states which are pivotal in their potential to transform into malignancy from normalcy [4]. The hallmarks described earlier in 2002 and modified in 2011 were the acquired ability of the cancer cells to sustain proliferative signalling, evade growth suppressors, resist cell death, replicative immortality, angiogenesis, invade and metastasise, reprogrammed the cellular metabolism, and evade immune destruction [5]. Currently apart the above mentioned 8 hallmarks, 4 more have been considered as emerging hallmarks which include enabling characteristics involving "unlocking phenotypic plasticity", "non-mutational epigenetic reprogramming", "polymorphic microbiomes", and "senescent cells". [4] .Though the advancement in science and drug development is enormous, the prognosis and treatment of OSCC continues to pose a major challenge to the medical fraternity. Hence there is a continuous thrive for development of novel genes and biomarkers which can be developed as potential targets for early diagnosis and therapeutic strategies not discounting their prognostic value. The present review highlights the significance of the FASTK family in health and disease (Figure 1 - see PDF) and hypothesises the molecular mechanism for its role in cancer (Figure 2 - see PDF).

## Mitochondrial protein functions:

Earlier quoted as an emerging hallmark and now included in the main component is the metabolic reprogramming of mitochondria is critical in cancer development [6]. Mitochondria contribute to cancer formation and progression by dysregulation of cell death, de-regulated metabolism, inflammation, genome instability and migration [7]. In Spite of the presence of mt-DNA (mitochondrial genome) which controls protein synthesis to an extent, the mitochondrial proteome is predominantly encoded by the nuclear genes and synthesised as precursor proteins on the cytosolic ribosomes [8]. Precursor proteins are: (i) targeted to mitochondria; (ii) imported into or across the outer membrane; and (iii) sorted into the correct sub-compartment. Such extensive protein import mechanism and sorting is executed by highly sophisticated translation machinery [8].

## FASTK Family:

Numerous protein families have been significant in maintaining mitochondrial organisation and stability which are crucial for disease prevention, one such family which is now under the radar is the Fas-activated serine/threonine kinase (FASTK) family of proteins that has emerged as key regulators in post-transcriptional gene expression in the mitochondria [9-11]. It contains six members: FASTK, the founding member, and its homologs FAST Kinase Domains 1-5 (FASTKD1-5) are expressed only in the mitochondrial matrix and hence regulate numerous functions in the mitochondria [9-11]. Their protein structure is composed of three domains called FAST 1, FAST 2, and RAP. Earlier studies predominantly concentrate on their functions in the mitochondrial RNA regulation, from mRNA processing and maturation to ribosome assembly and translation, but little is known about their role in disease [9-11]. A recent systematic analysis was performed in this family to elucidate their role in various cancers [12]. Apart from cancer they are also known to play roles in other diseases of mitochondrial and inflammatory origin. Proteins from the FASTK family therefore play crucial roles in health and disease states of the human body. Distinct role of FASTK family proteins in the regulation, processing and maturation of mitochondrial RNA in cancer, is still evasive.

## Physiological roles of FASTK family:

### FASTK:

The mechanism of action of FASTK proteins is under continuous research due to their unique structural characteristics. Mitochondrial FASTK isoform has been reported to be necessary for the biogenesis of the mitochondrial ND6 mRNA, which encodes an essential subunit of mitochondrial respiratory complex I[10]. The mitochondrial isoform of FASTK plays a negative regulatory role on nonopsonic phagocytosis of bacteria in macrophages through its action on mitochondrial respiratory complex I activity [13]. It effectively binds to RNA, and is a component of MRGs (mitochondrial RNA granules) which is activated during Fas-mediated apoptosis regulated by the phosphorylation of a nuclear RBP -TIA-1, and thus an important player in apoptosis [14].

### FASTKD1:

FASTKD1 controls the ND3 domain in the mitochondria and has a putative role in RNA stability-related processes. The down-regulation of mitochondrial MT-ND3 mRNA levels leads to decreased respiratory complex I abundance and activity [9]. Owing to its interaction with Cyclophilin D which is a part mitochondrial permeability transition (MPT) pore, FASTKD1 was proposed to be a regulator of mitochondrial-dependent cell death and survival. FASTKD1 protects cells from oxidative stress and ROS induced cell death, the mechanism of which remains unknown. It also modulates mitochondrial dynamics in a CypD-independent manner, as well as autophagy/mitophagy and caspase-3 activation C [15]. Owing to this function, FASTKD1 was considered as a novel target for modulation of oxidative stress induced cell death and post-myocardial infarction healing [16]. Apart from this, FASTKD1 is considered as an early precursor of hematopoietic cells. Hypomethylation in FASTKD1 was detected due to in utero tobacco exposure in a recent study proving its role in early development [17]. However there is a shortage of studies on its role in carcinogenesis.

### FASTKD2:

FASTKD2 has a unique role in mitochondrial respiration, RNA processing and translation and interacts with a defined set of mitochondrial transcripts including 16S ribosomal RNA (RNR2) and NADH dehydrogenase subunit 6 (ND6) messenger RNA [18]. FASTKD2-deficient cells reveal impaired cellular respiration with reduced activities of all respiratory complexes. Studies through RNA interference (RNAi) or by Clustered Regularly Interspaced Short Palindromic Repeats (CRISPR) reveals that FASTKD2 controls mitochondrial 16S rRNA and intra-mitochondrial translation. It is also largely implicated in the functioning of MRGs along with the other members and predominantly used to label them 19].

### FASTKD3:

FASTKD3 is another protein which localises to the mitochondrial matrix but is not expressed in the MRGs unlike the other members of the FASTK family but plays a role in controlling mRNA stability of various molecules including MT-ND2, MT-ND3, MT-CO2, MT-CYB, and MT-ATP8/6 and also essential for effective COX1 mRNA translation [20]. According to previous studies, targeted knockdown of FASTKD3 significantly reduced basal and maximal mitochondrial oxygen consumption and they were found to have a key role in mitochondrial respiration by modulating energy balance in cells exposed to adverse conditions by functionally coupling mitochondrial protein synthesis to respiration [9].

### FASTKD4:

Another interesting player in the FASTK family is FASTKD4 also called the TBRG4 (Transforming growth factor beta regulator) or cell cycle progression restoration protein 2 (CPR2) is a key regulator in mt-RNA functions and also implicated in numerous cancers. Activation of non-canonical processing site between MT-ND5 and MT-CYB may be involved with FASTKD4 protein [11]. Upon FASTKD4 depletion, the MT-ND5-CYB precursor accumulates, accompanied by a decrease in some mature transcript levels, including MT-ND5 and MT-CYB [11].The exact mechanism of TBRG4 in cancer is still under constant research. Even though it plays a significant role in the prognosis of various malignancies, its role in Oral Cancer remains elusive.

### FASTKD5:

FASTKD5 is a bonafide component of MRGs, and colocalizes with MRGs which differentially regulate the processing of mitochondrial RNAs and an essential component of protein synthesis [21]. Depletion of FASTKD5 resulted in an accumulation of unprocessed precursor RNAs and defective complex IV [21]. The literature on FASTKD5 is sparse and hence needs further evaluation and validation.

## Implication of FASTK family in Cancers:

FASTK family is an intriguing group of proteins who have a discernible role in cancer but their role in oral cancer is still unexplored. FASTK, FASTKD2 and FASTKD4 are upregulated in cancer tissue compared to healthy tissue or cell lines in numerous cancers as mentioned in the table (Table 1 - see PDF). Silencing of FASTK has decreased the proliferation and induced activation of the apoptotic pathway. Additionally, increased expression of FASTK in astrocytoma led to upregulated migration and invasion. These findings demonstrate that FASTK can be used as a prognostic biomarker but also as a therapeutic target [22].The other important FASTK proteins that have been implicated in tumour progression include, FASTKD2 and FASTKD4, already described in the Table. Knockdown of FASTKD4 lung cancer cells led to the inhibition of cell proliferation, migration, and invasion highlighting its role in tumour growth in lung cancer [23]. In the case of pancreatic adenocarcinoma, FASTK and FASTKD2 had also been involved in proliferation, invasion, and migration and hence poor prognosis [24,25]. Conversely, the overexpression of FASTKD3 in bladder cancer improved patient survival [26]. FASTK4 expression was detected in osteosarcoma cell lines. Its knockdown suppressed the proliferation, migration, and invasion of those cell lines [27].These effects were associated with a decrease in the expression of TGF-beta and PKT/AKT pathway. The results were comparable to a similar study in colorectal cancer. TBRG4 also differentially expressed with respect to 5-FU sensitivity and BEZ235-sensitive cell lines and was implicated in cell cycle progression and apoptosis. This shows that TBRG4 could portray therapeutic effects if silenced [28].Thus these proteins can be considered as potential prognostic markers but also as regulators of tumour growth in several types of cancer which has been illustrated (Figure 1 - see PDF).

## Mechanisms involved in cancer development:

### Role of mitochondria:

Mitochondrial biogenesis is an intriguing functional enigma in the cell as they are the only organelle which has a two way control, they possess their own genome, the mt-DNA, which requires dedicated gene expression machinery as well the control from the various nuclear encoded proteins which control and express within the mitochondria [29]. Mitochondrial proteins require a smooth coordination between the pathways controlling the mitochondrial protein production with the nucleo-cytoplasmic compartment for maintaining a harmonious environment [30]. Mitochondria are the energy powerhouse of the cell whose adequate function depends on the reciprocal interaction between two genomes, both mitochondrial and nuclear. The m-DNA encodes only 13 of the 1000s of proteins expressed in the mitochondria but any faulty expression in the mt-DNA encoded proteins leads to imbalance and hence disturbance in the electron transport chain and respiratory complexes which are indispensable for energy production and thus cell survival. The production of few RNA molecules is also influenced by mtDNA which has varied functions in the cytoplasm. Consecutively, any dysfunction in the mtDNA expression may lead to pathologies in humans [19-31].

### Mitochondrial protein pathway:

As mentioned earlier, many nuclear encoded proteins are essential for mitochondrial transcription, RNA processing, degradation and translation. Emerging players in the post-transcriptional mitochondrial gene expression regulation concerns members of the FASTK family of proteins, which also controls non-canonical RNA processing and are structurally related to RNA binding proteins (RBP) [32][33] Mitochondrial organisation is most importantly mediated by two components namely the transcription factors and RNA binding proteins (RBP) which regulate the mitochondrial gene expression and other functions like mRNA splicing, stability and translation respectively. RBPs can also undergo mutations and other alterations which lead to mitochondrial dysfunctions, constituting leading causes of pathogenesis (Figure 2 - see PDF).

In many cancers as already discussed, differential expression of FASTKs has been observed and some of them have been used as prognostic biomarkers. A large number of FASTKs have also been implicated in cancer development and in decreased drug sensitivity, suggesting that the FASTK family is a putative candidate for oncology research. Furthermore, some of these proteins act as tumour suppressors, by proapoptotic and increasing cell survival (e.g., FASTKD3) [9] while others act as pro-oncogenes and poor prognostic factors (FASTK, FASTKD2, FASTKD4) [25,27,28,34]. With the above findings we have formatted a possible role of FASTK family in the mechanism for tumorigenesis (Figure 2 - see PDF).

### Influencing factors in tumourigenesis:

There is increased recognition that genetic alterations affecting RNA splicing and polyadenylation are common in cancer and may generate novel therapeutic opportunities [35] Supek et al have identified variable sources for synonymous mutations in cancer, most of which are related to altered RNA splicing in oncogenes [36]. Recently, 'Epitranscriptome', a term defining post-transcriptional RNA modifications that can dynamically change the transcriptome was described [37]. They participate in modulating gene expression and controlling the cell fate, thereby instilling a quest for RNA-based drug discovery. Since the FASTK family of proteins have a major role in RNA processing and stability, mapping new RNA modifications, studying the biological functions of RNA modification-related genes and understanding their pathogenic mechanisms will be of prominence in the design and development of novel anticancer drugs [37]. Since the various cancers described are closely related to oral cancer developmental pathways, this review provides a whole new realm of proteins to be deliberated in the prognosis and treatment of oral cancer.

## Conclusion:

This review has concisely spotlighted the physiological and pathological roles of the relatively unexplored FASTK family and accentuated the mechanisms by which their functional alterations can lead to cancer development. Despite the substantial progress made in the field of mtRNA processing and its regulation, many concepts regarding their role in malignancies remain camouflaged. Our discussion is a sliver of the entire scope, with mitochondrial proteins engaging in a plethora of roles in almost every facet of cancer biology and thus optimistically unearths a whole new arena in drug design therapeutic strategies by imploring the roles of FASTK proteins, particularly in cancers.
